# The health of Saudi youths: current challenges and future opportunities

**DOI:** 10.1186/s12875-016-0425-z

**Published:** 2016-03-05

**Authors:** Maziar Moradi-Lakeh, Charbel El Bcheraoui, Marwa Tuffaha, Farah Daoud, Mohammad Al Saeedi, Mohammed Basulaiman, Ziad A. Memish, Mohammad A. Al Mazroa, Abdullah A. Al Rabeeah, Ali H. Mokdad

**Affiliations:** Institute for Health Metrics and Evaluation, University of Washington, 2301 Fifth Ave, Suite 600, Seattle, WA 98121 USA; Ministry of Health of the Kingdom of Saudi Arabia, Assadah, Al Murabba Riyadh 12613 Saudi Arabia

**Keywords:** Adolescent, Youth, Health status, Risk factors, Saudi Arabia

## Abstract

**Background:**

The health status of the young people is an important indicator for future health and health care needs of the next generation. In order to understand the health risk factors of Saudi youth, we analyzed data from a large national survey in the Kingdom of Saudi Arabia.

**Methods:**

The Saudi Health Information Survey sample included 2382 youths aged 15 to 24 years old. The questionnaire included information on socio-demographic characteristics, risk factors, risky behaviors, chronic conditions, functional status, health care utilization, and anthropometric and blood pressure measurements.

**Results:**

Only 45.9 % of men and 48.4 % of women had normal body mass index (BMI). Men were more likely than women to smoke cigarettes or shisha. The prevalence of daily consumption of at least five servings of fruits and vegetables was 6.6 %. The prevalence of no or insufficient physical activity was 41.8 % in men and 75.6 % in women (*P <* 0.001). Around 40 % of men and 25 % of women had abnormal blood pressure. Mean BMI and prevalence of insufficient physical activity, current smoking, and hypertension was higher in 20-to 24-year-olds than younger ages. Women were more likely to report that they never use seatbelts (82.2 % vs. 65.4 %).

**Conclusions:**

The prevalence of modifiable risk factors and risky driving behaviors is very high among Saudi youth. If these current behaviors are not reversed during this crucial age period, the burden of disease and injuries will rise in the future. Our findings call for developing health prevention programs for youths in Saudi Arabia.

## Background

The United Nations Secretariat defines youths as those aged 15 to 24 years old. The youth period is a time of transition from the dependence of childhood to the independence of adulthood [1]. The health status of the young people in a population is an important indicator for future health and health care needs of the next generation. Patton et al. argued that there are good opportunities for health gains both through prevention and early clinical intervention in this period of life. They recommended a list of indicators for monitoring health status of youth and adolescence [2]. Many of the preventable risky behaviors, such as misuse of alcohol, tobacco, drugs, unsafe sex, unsafe driving, and violence, are usually established during the youth period [3]. Indeed, youth is an important formative period for patterns of behavior that endure into adulthood [4].

The Kingdom of Saudi Arabia (KSA) has a young population; more than half of the population is younger than 25 years, and approximately 14 % of Saudis are aged 15 to 24 years old [5]. The KSA burden of disease, injuries, and risk factors study showed that mental and behavioral disorders, injuries, and other non-communicable diseases are the leading causes of disease burden in Saudi youths [6]. Few other studies have reported specific aspects of the health of young people in KSA, such as overweight and obesity [7–9], nutritional status [10–13], or specific diseases. Moreover, most of the previous studies are not generalizable to all Saudi youths due to limitations to specific geographical areas (such as a province or city) or to specific populations (such as students). In order to describe the current status of diseases, health risk factors, and healthcare utilization among Saudi youths (15-to 24-year-old individuals), we analyzed data from a large national household survey in KSA.

## Methods

The Saudi Health Information Survey (SHIS) was a cross-sectional national multistage survey of individuals aged 15 years or older performed between April and June 2013. For the purpose of this survey, KSA was divided into 13 regions, and each region was divided into subregions and blocks. All regions were included, and a probability proportional to size method was used to randomly select subregions and blocks. Households were randomly selected from each block. A roster of household members was conducted, and one individual aged 15 or older was randomly selected from the household to be surveyed. If the randomly selected person was not present, surveyors made another appointment to return, up to three times, before considering the household as a nonresponse. For the current study, we selected the subsample of 15-to 24-year-old youths from the SHIS dataset. More details on the methods and other findings of this survey are available in previously published reports [14–20].

### Ethics, consent and permissions

The Saudi Ministry of Health and its Institutional Review Board (IRB) approved the study protocol. The University of Washington IRB deemed the study as IRB-exempt, since the Institute for Health Metrics and Evaluation received de-identified data for this analysis. All respondents consented and agreed to participate in the study. If the respondent was between the ages of 15 and 17 years old, the parent (s) or legal guardian of that individual consented as well.

Trained local health professional staff performed the interviews with same-sex respondents.

The survey included questions on socio-demographic characteristics, a selected list of risk factors, chronic conditions, functional status, and health care use.

We measured self-rated health by asking respondents “In general, would you say your health is excellent, very good, good, fair, or poor?” with a per-question explanation about its timeframe during the past 30 days.

Individuals were classified into three groups of never smokers, former smokers, and current smokers through two questions: “Have you ever smoked any tobacco products, such as cigarettes, cigars or pipes, or shisha?” and “Do you currently smoke any tobacco products, such as cigarettes, cigars, pipes or shisha?” Interviewers also asked respondents about daily use of shisha, pipe, or flavored shisha, which was considered as daily shisha smoking.

A total of three blood measurements were taken with the participant resting and at five-minute intervals. The guidelines of the National Health and Nutrition Examination Survey (NHANES) were used for measuring blood pressure levels [21]. Individuals were classified based on measures of systolic (SBP) and diastolic blood pressure (DBP) as having: normal blood pressure (SBP ≤ 120 and DBP ≤ 80), pre-hypertension (120 < SB*P <* 140 or 80 < DB*P <* 90), Stage 1 hypertension (140 ≤ SB*P <* 160 or 90 ≤ DB*P <* 100), and Stage 2 hypertension (SBP ≥ 160 or DBP ≥ 100).

Trained interviewers measured weight and height to calculate body mass index (BMI) as kg/m^2^. Participants were classified into four groups: underweight (BMI < 18.5), normal weight (18.5 ≤ BMI < 25), overweight (25 ≤ BMI < 30), and obese (BMI ≥ 30).

We used the short form of the International Physical Activity questionnaire to measure physical activity in occupational and recreational settings. The respondents were classified into four groups: met vigorous physical activity, met moderate physical activity, insufficient physical activity to meet vigorous or moderate levels, and no physical activity [22].

Respondents were asked, “In a typical week, how much time do you usually spend in front of the television or on the computer?” Respondents were also asked to rate their ability to perform activities. They were asked for their ability to perform vigorous activities (“Does your health now limit you in doing vigorous activities, such as running, lifting heavy objects, or participating in strenuous sports?”), mild activities (“During the past 30 days, how difficult was it to perform any of the following activities: walking a short distance, standing from a seated position, standing for a short period of time, climbing one step of stairs?”), their usual work or house activities (“During the past 30 days, how difficult was it to perform your work or house activities?”), and specific functional abilities (“Are you able to climb up five steps?”). Interviewers also asked whether or not the respondent required the use of special equipment such as a cane, a wheelchair, a special bed, or a special telephone.

We computed the servings of fruits and vegetables consumed per day from a detailed dietary questionnaire as the sum of the average daily servings. Respondents were asked, “In a typical week, how many days do you eat processed meats such as sausage, or other packaged cold cuts, lunch meats, or deli meats?” and “How many servings of processed meats do you eat on one of those days?” Similar questions were asked for “other processed foods, such as fast foods, canned foods, packaged entrees, or soup,” “fast foods,” and “regular soda or pop that contains sugar, sweetened iced teas, sports drinks, or fruit drinks.”

To assess diagnosed cases of asthma, diabetes mellitus (and type of diabetes), congestive heart failure, renal failure, and cancer, interviewers asked, “Has a doctor or other health professional ever told you that you had [that condition]?” Questions about talking on a mobile or using a handset or hands-free device while driving, following speed limits, and using seatbelts in different settings of driving or traveling in a car were asked in order to assess driving safety.

We used stata 13.1 for Windows (StataCorp LP, TX, USA) for survey analysis. Data were weighted to account for the probability of selection and age and sex post-stratification. We used individual sampling weights to account for the probability of selecting a respondent within a household, the probability of selecting a household within a stratum, and the post-stratification differences in age-and sex-distribution between the Saudi population and the sample.

Descriptive data are reported as percent and standard error (SE) for categorical variables or mean and SE for numerical variables. We used stepwise multivariate logistic regression. We used Chi-square and pooled t tests to compare measures between subcategories of sex or between younger (15-to 19-year-old) and older (20-to 24-year-old) youths.

## Results

There were 2382 individuals 15 to 24 years old in the SHIS database (response rate for SHIS: 89.4 %). Table 1 describes the socio-demographic characteristics of this sample. About 14 % of female and 3 % of male youths reported a history of marriage. Among youths under the age of 18, 1.9 % (*n =* 7) of boys and 3.9 % (*n =* 20) of girls reported a history of marriage; these percentages were 3.2 % and 13.8 % among all male and female youths, respectively. College education (any education after completing high school) was higher among women than men (*P <* 0.001), however, the percentage of current (school or university) students was higher in men. The percentage of men who had a paid job was twice that of women. Approximately 27 % of youth classified as “other” work status (including those who had unpaid jobs, were unemployed or unable to work, and homemakers) had college or higher education; the percentage of this other work status was 8.1 times higher for women than for men.

**Table 1:** Socio-demographic characteristics of Saudi youths by sex, 2013

Factor	Categories	Male			Female			Both sexes		
		N	Weighted %	SE	N	Weighted %	SE	N	Weighted %	SE
Age	15–19 years	677	58.6	1.8	547	48.5	1.9	1224	53.7	1.3
	20–24 years	512	41.4	1.8	646	51.5	1.9	1158	46.3	1.3
Education	Primary school or less	153	14.5	1.3	175	14.9	1.4	328	14.7	0.9
	Elementary/high school	941	76.5	1.6	824	68.4	1.8	1765	72.6	1.2
	College or higher education	94	9.0	1.1	192	16.7	1.4	286	12.8	0.9
Marital status	Never married	1135	96.8	0.6	925	86.2	1.1	2060	91.6	0.6
	Currently married	53	2.9	0.5	252	12.3	1.0	305	7.5	0.6
	Separated, divorced, widowed	1	0.3	0.3	16	1.6	0.5	17	0.9	0.3
Work status	Employed	145	10.9	1.1	65	5.5	0.9	210	8.2	0.7
	Student	933	80.6	1.4	795	70.7	1.7	1728	75.8	1.1
	Unemployed	96	6.7	0.9	112	9.0	1.1	208	7.8	0.7
	Other	15	1.8	0.6	221	14.9	1.3	236	8.2	0.7

Self-rated health did not vary by age. About 65 % of respondents reported that their health was excellent. Less than half of respondents had normal BMI (18.5-24.9 kg/m^2^). Underweight, overweight, and obesity did not vary significantly by sex. Prevalence of overweight or obesity in men and women was 38.2 % and 43.9 %, respectively. BMI increased with age from 23.5 (±0.2) in those aged 15 to 19 years to 25.1 (±0.2) kg/m^2^ in those 20 to 24 years old (*P <* 0.001). Men were more likely than women to smoke cigarettes (16.1 % current smoking in men versus 0.8 % in women) or shisha (5.0 % daily shisha smoking in men versus 1.0 % in women). The average pack years of smoking for current smokers was 3.43 (SE = 0.42) for men and 0.12 (SE = 0.08) for women. Smoking was much higher in the older age group (Table 3).

The prevalence of daily consumption of at least five servings of fruits and vegetables was low in both sexes (6.6 %) and did not vary by age (Table 2). Men reported higher levels of physical activity than women (Table 2); 19.7 % of men and 42.8 % of women were classified as inactive based on their reported levels of physical activity. Overall, physical activity was very low and even lower among older youth (Table 3). Men were more likely to consume fast food and processed food (Table 2). Around 7.0 % (*SE* = 0.9) of men and 14.9 % (*SE* = 1.3) of women reported that they never drink soda or pop in a regular week. Consumption of processed food (other than meat) was marginally higher in the older ages (Table 3). Women were more likely to report never using seatbelts as passengers than men (82.2 % vs. 65.4 %). Younger males were more likely to report using handset cellphones and less likely to use seatbelts than older males. High blood pressure and diagnosed cases of asthma were more common in male youth. Those aged 20 to 24 years were more likely to have high blood pressure (Tables 2 and 3).

**Table 2:** Risk factors of health among Saudi youths by sex, 2013

Factor	Categories	Male			Female			*P* value
		N	Percent/Mean	SE	N	Percent/Mean	SE	
BMI (%)	<18.5 Kg/m^2^	164	14.4	1.3	131	12.3	1.3	0.564
	18.5-24.9 Kg/m^2^	544	47.5	1.9	568	50.5	1.9	
	25-29.9 Kg/m^2^	284	24.9	1.6	272	23.4	1.6	
	30+ Kg/m^2^	157	13.3	1.3	170	13.9	1.3	
Smoking (%)	Never smoked	943	81.2	1.5	1169	98.5	0.5	<0.001
	Former smoker	42	2.7	0.6	8	0.8	0.4	
	Current smoker	202	16.1	1.4	11	0.8	0.3	
Shisha (%)	Daily users	60	5.0	0.8	15	1.0	0.4	<0.001
	Others	1113	95.0	0.8	1178	99.0	0.4	
Fruit and vegetables (%)	No servings	407	37.5	1.8	442	39.0	1.8	0.073
	1 to 4 servings	718	57.2	1.8	683	53.0	1.9	
	5 or more servings	64	5.3	0.8	68	8.0	1.1	
Activity (%)	No physical activity	255	19.7	1.4	532	42.8	1.9	<0.001
	Insufficient	245	22.1	1.6	346	32.8	1.8	
	Moderate	177	16.1	1.4	113	9.0	1.0	
	Vigorous	479	42.1	1.9	174	15.4	1.4	
Blood pressure (%)	Normal blood pressure	741	64.38	1.81	908	79.54	1.54	<0.001
	Prehypertension	362	31.26	1.76	218	18.00	1.45	
	Stage 1 hypertension	44	3.93	0.74	18	2.00	0.60	
	Stage 2 hypertension	5	0.43	0.24	5	0.46	0.24	
Daily sitting time (hr)		1129	4.4	0.1	1113	4.7	0.1	0.022
Weekly TV and computer time (hr)		1137	7.8	0.4	117	7.9	0.4	0.874
Days eating fast food (per week)		1080	2.6	0.1	1057	1.9	0.1	<0.001
Soda or pop^a^ (numbers per week)		1064	2.2	0.1	968	2.5	0.2	0.180
Days eating processed meat (per week)		1120	0.7	0.1	1098	0.6	0.0	0.176
Processed meat (servings per day)		310	1.6	0.1	296	1.5	0.1	0.243
Days eating other processed food (per week)		1125	1.6	0.1	1113	1.4	0.1	0.030
Other processed food (servings per day)		674	1.6	0.1	633	1.4	0.1	0.007
Uses seatbelt as front passenger (%)	Never	789	65.4	1.8	949	82.2	1.4	<0.001
	Sometimes	323	30.3	1.7	196	16.1	1.4	
	Always	59	4.4	0.7	20	1.7	0.5	
Uses seatbelt as back passenger (%)	Never	1039	89.1	1.1	1079	91.7	1.1	0.238
	Sometimes	111	9.9	1.1	81	7.6	1.0	
	Always	23	1.0	0.2	9	0.7	0.3	

^a^Carbonated soft drinks

**Table 3:** Comparison of risk factors of health in 15 to 19 year olds and 20 to 24 year olds in Saudi Arabia, 2013

Factor	Categories	15-19 years			20-24 years			*P* value
		N	Percent/Mean	SE	N	Percent/Mean	SE	
BMI (%)	<18.5 Kg/m^2^	196	17.2	1.4	99	8.9	1.1	<0.001
	18.5-24.9 Kg/m^2^	601	50.4	1.8	511	47.2	2.0	
	25-29.9 Kg/m^2^	242	20.9	1.5	314	28.0	1.7	
	30+ Kg/m^2^	141	11.6	1.2	186	15.9	1.4	
Smoking (%)	Never smoked	1148	94.4	0.9	964	84.0	1.4	<0.001
	Former smoker	26	2.1	0.5	24	1.4	0.4	
	Current smoker	49	3.5	0.7	164	14.6	1.4	
Shisha (%)	Daily users	19	1.5	0.4	56	4.8	0.9	<0.001
	Others	1199	98.5	0.4	1092	95.2	0.9	
Fruit and vegetables (%)	No servings	462	39.8	1.8	387	36.6	1.9	0.472
	1 to 4 servings	694	53.9	1.8	707	56.5	1.9	
	5 or more servings	68	6.3	0.9	64	6.9	1.1	
Activity (%)	No physical activity	325	25.1	1.6	462	37.7	1.9	<0.001
	Insufficient	321	29.0	1.7	270	25.4	1.7	
	Moderate	155	12.6	1.2	135	12.7	1.3	
	Vigorous	389	33.3	1.7	264	24.2	1.7	
Blood pressure (%)	Normal blood pressure	909	77.2	1.56	740	65.45	1.88	<0.001
	Prehypertension	251	20.8	1.51	329	29.55	1.80	
	Stage 1 hypertension	21	1.68	0.45	41	4.53	0.89	
	Stage 2 hypertension	6	0.43	0.21	4	.46	0.27	
Daily sitting time (hr)		1155	4.5	0.1	1087	4.6	0.1	0.444
Weekly TV and computer time (hr)		1165	7.6	0.3	1089	8.1	0.4	0.360
Days eating fast food (per week)		1113	2.2	0.1	1024	2.3	0.1	0.459
Soda or pop^a^ (Numbers per week)		1074	2.3	0.1	958	2.4	0.2	0.696
Days eating processed meat (per week)		1157	0.6	0.05	1061	0.7	0.1	0.204
Processed meat (servings per day)		304	1.6	0.1	302	1.5	0.1	0.511
Days eating other processed food (per week)		1160	1.4	0.1	1078	1.6	0.1	0.049
Other processed food (servings per day)		679	1.5	0.05	628	1.6	0.1	0.073
Uses handset cell phone while driving^b^ (%)	Never	100	21.3	2.4	64	11.4	1.7	0.003
	Sometimes	329	70.1	2.7	362	75.5	2.6	
	Always	40	8.6	1.7	46	13.1	2.3	
Uses hands-free cell phone while driving^b^ (%)	Never	348	72.7	2.6	326	68.4	2.8	0.439
	Sometimes	109	23.4	2.5	127	28.0	2.7	
	Always	19	3.9	1.0	19	3.6	1.1	
Follows speed limits^b^ (%)	Never	113	24.1	2.5	95	20.6	2.4	0.387
	Sometimes	237	51.5	3.0	279	57.3	3.0	
	Always	103	24.4	2.7	93	22.1	2.6	
Uses seatbelt while driving^b^ (%)	Never	299	60.6	2.9	239	48.6	3.0	0.007
	Sometimes	155	34.6	2.8	206	46.9	3.0	
	Always	24	4.8	1.2	28	4.4	1.0	
Uses seatbelt as front passenger (%)	Never	917	75.3	1.6	821	71.4	1.8	0.248
	Sometimes	253	21.9	1.5	266	25.1	1.7	
	Always	33	2.8	0.7	46	3.5	0.6	
Uses seatbelt as back passenger (%)	Never	1098	90.6	1.1	1020	90.2	1.1	0.800
	Sometimes	91	8.5	1.1	101	9.1	1.0	
	Always	16	0.9	0.3	16	0.7	0.2	

^a^Carbonated soft drinks

^b^Only men answered this item

The prevalence of diagnosed diabetes mellitus was not different by sex (0.8 % in male and 0.9 % in female). There were no significant differences in prevalence of diagnosed diabetes mellitus between those aged 15 to 19 years and those aged 20 to 24 years.

Women were more likely to report having limitations in performing vigorous physical activities (24.7 % versus 12.6 %) and usual housework activities (9.8 % versus 5.2 %) than men. Around 0.17 % of respondents needed special equipment due to health problems. Deafness or trouble in hearing was reported by 3.0 % of men compared to 1.5 % of women. However, there were no significant difference between those aged 15 to 19 years and those aged 20 to 24 years (Table 4).

**Table 4:** Indicators of physical functioning in Saudi youths by sex, 2013

Factors	Categories	Male			Female			*P* value
		N	Percent	SE	N	Percent	SE	
Limitation in vigorous physical activity	No	1032	87.4	1.2	871	75.3	1.7	<0.001
	Yes	145	12.6	1.2	272	24.7	1.7	
Need for special equipment	No	1176	99.7	0.1	1183	99.9	0.0	0.056
	Yes	7	0.3	0.1	2	0.1	0.0	
Able to climb five steps?	Without any difficulty	1148	96.0	0.8	1099	94.7	0.8	0.164
	With a little difficulty	26	2.3	0.5	60	4.1	0.7	
	With some difficulty	11	1.5	0.6	10	1.1	0.4	
	With much difficulty	2	0.1	0.0	2	0.1	0.1	
	Unable to do	2	0.1	0.1	0	0.0	_	
Difficulty in performing house/work activities?	Without any difficulty	1100	94.8	0.9	1018	90.2	1.1	0.007
	With a little difficulty	40	3.9	0.8	102	7.1	0.9	
	With some difficulty	9	1.0	0.5	27	2.5	0.6	
	With much difficulty	3	0.3	0.2	4	0.2	0.1	
	Unable to do	2	0.1	0.1	0	0.0	_	
Wears glasses or contact lens	No	1050	89.4	1.1	1027	85.1	1.3	0.016
	Yes	133	10.6	1.1	162	14.9	1.3	
Far vision	No difficulty	1077	91.6	1.0	1049	88.9	1.2	0.342
	Some difficulty	105	8.4	1.0	133	11.1	1.2	
Near vision	No difficulty	1119	95.3	0.7	1098	92.7	1.0	0.076
	Some difficulty	63	4.7	0.7	86	7.3	1.0	
Deafness or trouble hearing	No	1148	97.0	0.7	1164	98.6	0.4	0.022
	Yes	39	3.0	0.7	27	1.5	0.4	

Use of health care services was not significantly different between men and women. A notable proportion of respondents (46.2 % of men and 44.1 % of women) had visited a hospital for medical attention in the past 12 months. Injury was the reason for visiting a hospital in 11.7 % (*n =* 80) of men compared to 2 % (*n =* 17) of women. Injury was also a more common reason for visiting a hospital for those aged 15 to 19 years than for those aged 20 to 24 years (8.8 % versus 4.8 %). Outpatient clinic and physician office visits were less frequently reported than hospital visits (Table 5). As for hospital visits, injury was a more common reason for males than females to visit an outpatient clinic (7.6 % versus 1.7 %) or a physician office (9.4 % versus 1.9 %). Both routine medical checkups and oral examinations were more common in those aged 20 to 24 years compared to those aged 15 to 19 years (Table 5).

**Table 5:** Comparison of health care use between 15 to 19 year olds and 20 to 24 year olds in Saudi Arabia, 2013

Health care	Time range	15-19 years			20-24 years			*P* value
		N	Percent	SE	N	Percent	SE	
Routine checkup	Within 12 months	84	7.3	1.0	118	10.6	1.2	<0.001
	1 to 5 years	64	5.2	0.8	108	9.4	1.1	
	Never or >5 yr earlier	993	87.5	1.3	861	80.0	1.5	
Hospital	Within 12 months	481	46.7	1.9	460	43.4	2.0	0.347
	1 to 5 years	216	17.7	1.5	224	20.5	1.6	
	Never or >5 yr earlier	427	35.6	1.8	386	36.1	1.9	
Outpatient clinic	Within 12 months	499	46.1	1.9	451	39.7	2.0	0.045
	1 to 5 years	195	15.8	1.4	200	19.5	1.6	
	Never or >5 yr earlier	416	38.1	1.8	401	40.8	2.0	
Physician office visit	Within 12 months	483	42.8	1.9	455	42.0	2.0	0.787
	1 to 5 years	158	12.7	1.3	143	14.0	1.5	
	Never or >5 yr earlier	449	44.5	1.9	427	44.0	2.0	
Oral examination	Within 12 months	103	9.1	1.1	151	13.2	1.3	0.014
	Never or >1 yr earlier	1112	90.9	1.1	996	86.8	1.3	

## Discussion

In a national study in KSA, we showed a high prevalence of health risk factors (overweight and obesity, low physical activity, low consumption of fruits and vegetables, high prevalence of high abnormal blood pressure) in Saudi youth. We found that these risk factors are increasing with age and are higher in older youths. Furthermore, we demonstrated that youth are engaging in highly risky behaviors while driving. These findings are of great concern for the KSA health authorities. Our findings call for developing and implementing youth-appropriate programs to reduce the burden of disease and to avoid future health challenges.

Although prevalence of overweight and obesity has been previously reported as higher in females compared to males in Saudi adolescents [23] and Saudi adults [17], the prevalence was not considerably different between men and women in our study. This might be due to the decreasing trend of obesity in younger age cohorts of both sexes, which is more prominent in women [17]. Prevalence of obesity was similar to adolescents in the United States (13.7 %), but overweight in our sample (24.1 %) was more common compared to the US adolescent population (16.6 %) [24]. Current smoking among US high school adolescents is higher than in Saudi Arabia (15.7 % versus 8.6 %); prevalence of current smoking in US men and women is similar (16.4 % and 15.0 %, respectively), while it is very different in KSA (16.1 % in men and 0.8 % in women) [24].

Consumption of fast food and soda or sweetened beverages was high among Saudi youth. Those who never drink soda or pop in KSA were less than US adolescents (10.9 % vs. 22.3 %, respectively) [24]. There was a combination of low physical activity, overweight and obesity, and unhealthy diet in many of the Saudi youths in our study; Washi et al. reported a non-healthy diet with high carbohydrate and fat intake and low calcium, iron, and zinc intake in Saudi adolescents [13]. Pearson and Biddle showed a strong association between sedentary behavior and unhealthy diet [25]. In Saudi Arabia, dietary risk factors, high BMI levels, and high blood sugar levels are amongst the leading causes of disability-adjusted life years [6]. The fact that these risk factors are prevalent at younger ages deserves immediate attention and intervention. Low physical activity was much more prevalent in female youth; public health authorities should push all related sectors to facilitate access for women to health facilities and encourage an active lifestyle for women.

Injuries, especially transport injuries, are among leading causes of mortality and morbidity among youths, both worldwide and in Saudi Arabia [6, 26]. We observed a high prevalence of risky driving and traveling behaviors in Saudi youth. Around three-quarters of young drivers reported that they do not follow speed limits. Moreover, most of the studied Saudi youths reported that they do not use seatbelts as a driver or as a passenger; the percentage of reporting never use of seatbelt as passenger in KSA was 73.5 %, compared to 7.6 % in US adolescents [24]. The prevalence is likely to be even higher in reality, as some youth may not report this illegal behavior to interviewers. There is an immediate need for law enforcement by relevant authorities and proper education around the importance of safe driving and traveling behaviors [15].

Hospital/emergency room visits were high among both male and female youths. In fact, they were reported more than routine medical checkups and oral health examinations. This practice is not sustainable, even in a wealthy country like KSA. Clearly, many youth are experiencing health problems that they ignore until they need to seek emergency or hospital care. The fact that health care is free in KSA calls for the Ministry of Health to encourage the use of preventive care rather than emergency care. A message to educate and inform youth about the value of preventive care is urgently needed, perhaps through school, as the majority of Saudis attend school [27]. On the other hand, we did not collect further data about the reasons for emergency room or hospital visits; some of these visits might be due to non-urgent problems that can be addressed through outpatient visits.

The unemployment rate among youth in our study was lower than what others have previously reported [5]. However, with KSA’s young population, more jobs will be required in the near future. Our study showed that women were less likely to be employed than men. Other studies have explained a gender-based mismatch between education and job opportunities in the Arab world [28]. Future employment plans should take into account the growing population of educated women that will enter the market. Marital status is one of the important demographic indicators that affects social, mental, and physical health. In a country with Islamic rules such as Saudi Arabia, marriage is one of the very few socio-culturally acceptable ways of having a sexual relationship. It potentially affects education, employment, and fertility in the following years.

There is a growing evidence base for action to improve the health of adolescents and young people [29]. The interventions might target diseases, proximal risk factors, or determinants of health. Such interventions will influence incidence and severity of diseases (especially non-communicable diseases) and injuries at older ages as well as affecting the health status of the current youth population [3, 30]. As part of related interventions, health care providers need to achieve core competencies for appropriate approach to and management of health and development problems of young people; specific attitudes, knowledge, and skills are required for working with youth [31]. The Ministry of Health should ensure that providers are trained in such skills.

This study has some limitations. First, our data are cross-sectional, hence we cannot assess causality. Second, many of our behavioral data, such as smoking, seatbelt use, or physical activity, are self-reported and subject to recall and social desirability biases. Despite these limitations, our study is based on a large sample size and used a standardized methodology for all its measures. We extracted all indices related to adolescence and youth health available through the SHIS data [2], however, there were not enough data on some important aspects of youth health such as sexual behaviors, violence, mental health, and alcohol or drug use.

## Conclusion

The prevalence of health risk factors and risky driving behaviors are very high among Saudi youths. If these current behaviors are not reversed during this crucial age period, the burden of disease will increase in KSA in coming years. Our findings call for immediate interventions to reverse these trends. Youth is an important phase in our lives. It has many challenges and requires more attention and resources for intervention. However, along with the challenges are great opportunities for the future, and changes made in this generation will shape the future health and well-being in the Kingdom. Our findings call for a focused program for youth health in KSA. Prevention should be at the forefront of all activities for better youth health and future health of the Kingdom.
